# Spatial Analysis of Groundwater Hydrochemistry through Integrated Multivariate Analysis: A Case Study in the Urbanized Langat Basin, Malaysia

**DOI:** 10.3390/ijerph18115733

**Published:** 2021-05-27

**Authors:** Nur Fatihah Mohamad Zainol, Azim Haziq Zainuddin, Ley Juen Looi, Ahmad Zaharin Aris, Noorain Mohd Isa, Anuar Sefie, Ku Mohd Kalkausar Ku Yusof

**Affiliations:** 1 Department of Environment, Faculty of Forestry and Environment, Universiti Putra Malaysia, Serdang 43400 UPM, Malaysia; nurfatihah.mzainol@gmail.com (N.F.M.Z.); leyjuenlooi@upm.edu.my (L.J.L.); 2 International Institute of Aquaculture and Aquatic Sciences, Universiti Putra Malaysia, Port Dickson 71050, Malaysia; azimhazeeq@gmail.com (A.H.Z.); zaharin@upm.edu.my (A.Z.A.); 3 National Water Research Institute of Malaysia, Lot 5377, Jalan Putra Permai, Seri Kembangan 43300, Malaysia; anuar@nahrim.gov.my; 4 Department of Chemistry, Universiti Malaysia Terengganu, Kuala Terengganu 21300, Malaysia; kukausar@umt.edu.my

**Keywords:** groundwater assessment, groundwater quality, multivariate statistical analysis, heavy metals, major ions, weathering

## Abstract

Rapid urbanization and industrial development in the Langat Basin has disturbed the groundwater quality. The populations’ reliance on groundwater sources may induce possible risks to human health such as cancer and endocrine dysfunction. This study aims to determine the groundwater quality of an urbanized basin through 24 studied hydrochemical parameters from 45 groundwater samples obtained from 15 different sampling stations by employing integrated multivariate analysis. The abundance of the major ions was in the following order: bicarbonate (HCO_3_^−^) > chloride (Cl^−^) > sodium (Na^+^) > sulphate (SO_4_^2−^) > calcium (Ca^2+^) > potassium (K^+^) > magnesium (Mg^2+^). Heavy metal dominance was in the following order: Fe > Mn > Zn > As > Hg > Pb > Ni > Cu > Cd > Se > Sr. Classification of the groundwater facies indicated that the studied groundwater belongs to the Na-Cl with saline water type and Na-HCO_3_ with mix water type characteristics. The saline water type characteristics are derived from agricultural activities, while the mixed water types occur from water–rock interaction. Multivariate analysis performance suggests that industrial, agricultural, and weathering activities have contributed to groundwater contamination. The study will help in the understanding of the groundwater quality issue and serve as a reference for other basins with similar characteristics.

## 1. Introduction

Many parts of the world are experiencing a shortage of water supply; hence, groundwater acts as a substitute for the lack of available surface water. In most cases, groundwater possesses a valuable property in comparison to surface water. Half of the world’s total potable water is pumped from groundwater aquifers, with the highest dependency on groundwater consumption occurring in megacities [1].

Following the rapid pace of economic growth and coupled with pressures from an increasing population, development has inevitably encroached into groundwater areas. This encroachment is further exacerbated by the rising demands of the manufacturing industry, along with the usage of chemicals and the never-ending demand for food, both locally and globally, that has led to a tremendous increase in the application of fertilizers and pesticides [2]. In many developing countries, improper disposal of wastewater coming from municipal, industrial, and agricultural sources with little to no treatment before discharge is considered a common practice, and this has led to the eventual leaching of contaminants into the soil, which causes the significant depletion of groundwater quality [3]. All of these, in addition to unexpected climate change and the indiscriminate use of groundwater, have put pressure on groundwater resources in various aspects and created an imbalance in groundwater hydrology [4]. These contaminations will deteriorate the groundwater quality, which makes it unsafe to be used and results in adverse health effects.

Owing to the slow movement of groundwater flow, polluted groundwater takes a very long time to purify as it takes a more extended period to flush out the contaminants. Groundwater quality and its characteristics hugely depend on the nature of the soil through which the water percolates, as well as the rock matrix comprising the aquifer. Chemical characteristics such as groundwater mineralogical composition; contact time between the groundwater and its surroundings; the chemical phases through the rock formation process; water temperature and pressure; and other factors including its pH, the presence of organic matter, and dissolved oxygen can be useful in determining the degree of contamination in a groundwater system [5].

Malaysia’s total groundwater reserve is reported to be 5000 billion cubic meters, which is nine times the volume of water flowing into Malaysian rivers annually [6]. Around 70% of the total groundwater usage in Malaysia is served for domestic purposes, 25% for industrial usage, and the remainder for agricultural uses. In 2008, the extraction of groundwater in Selangor alone was estimated to be approximately 10.8 billion liters, with more than 300 active tube wells recorded, most of which could be found in schools and mosques, as well as in industrial and rural areas [7]. The demand for groundwater usage has increased in this country due to several reasons such as the lower cost and effort required for its extraction, causing many factories and other consumers to start to shift their freshwater sources to groundwater [8]. Apart from that, potable water is a critical component for human survival. Hence, its dependence should not only focus on surface water sources for meeting this requirement but also on alternative water sources such as groundwater.

Despite the rising cases of groundwater contamination and the proven importance of clean groundwater resources, research exploration focusing on Malaysian groundwater contamination is still scarce, while it is crucial for evaluating the quality and suitability of groundwater for various utilitarian purposes [9]. This is in accordance with the Strategies to Enhance Water Demand Management in Malaysia under strategy 19, which highlighted several points for groundwater, which include undertaking detailed groundwater assessment studies to develop and maintain the groundwater for use in emergencies and in major urban areas in relatively dry regions [10]. The necessity of this study is aligned with sustainable development goals (SDG), which ensure the availability and sustainable management of water and sanitation for all. Aside from that, most groundwater studies reported in Malaysia show a focus on island-based discussion, while 40% of groundwater studies focus on inland study areas. Table 1 summarizes the available inland groundwater contamination studies in Malaysia. From that, it is observed that contamination concentrated around landfill areas has become a popular subject for inland groundwater contamination studies; moreover, the previous researchers have discussed more about geophysical investigation. The lack of comprehensive hydrogeochemical studies in these studies has resulted in a limited understanding of groundwater chemistry variation, where additional factors such as intensive agriculture in coastal areas and excessive chemical fertilizer application could have a negative impact on water quality. Groundwater chemical composition alone may not be able to distinguish between natural and anthropogenic factors. The exploration of groundwater contamination involves processing a variety of parameters and factors leading to a complex and broad data collection [11]. Hence, the identification of the mechanisms and the determination of the most critical factors responsible for hydrogeochemistry require a powerful data interpretation tool. Through modern approaches such as multivariate analysis, it is possible to identify the variables that affect the outcomes, thereby offering a more accurate conclusion including apportioning pollution sources/factors (natural or anthropogenic), designing a monitoring network for effective water resource management, and finding practical solutions to pollution problems. This current study shows the relevance of this research application and will eventually fill the existing gap in evaluating the factors contributing to the pollution of groundwater.

It is necessary to conduct a comprehensive groundwater quality analysis, as it can help to identify the sources of the factors that influence groundwater quality, and the results can be used as reference data and initial guidance for decision-making and mitigation schemes for pollution control and technology by the relevant authorities [26,27,28]. The use of spatial analysis has made assessing environmental issues, such as groundwater, much easier [29]. Furthermore, the outcomes of this study will provide the information used to alert society to the importance of maintaining a pollutant-free groundwater system. Hence, the aims of the study are (i) to demonstrate the variation in the hydrogeochemistry of the Langat basin and (ii) to identify the most significant factor or source that influences the groundwater quality along the Langat Basin through a statistical approach. This study attempts to elucidate the spatial distribution of the groundwater quality of the Langat Basin along with the groundwater contamination. The results will later be compared with the appropriate national or international standard to ascertain its suitability as a water source.

## 2. Materials and Methods

### 2.1. Study Area

The Langat Basin is located on the west side of Peninsular Malaysia. It is a flat lowland with a catchment area of 1815 km^2^. The estimated total area of the Langat Basin is 2350 km^2^. The basin is drained by four large sub-basins, which are the Langat River, the Labu River, the Semenyih River, and the Beranang River, which flow west to the Strait of Melaka. The basin is among one of the most urbanized basins in Malaysia and is a highly developed urban zone. The Langat Basin consists of the Kuala Langat, Ulu Langat, and Sepang districts. In 1995, the population of the Langat Basin was 1,050,990, and this number has risen significantly over the years, with the total population of the studied area in 2019 being reported as 1,928,000 [30]. The increasing trend in the population has occurred due to several factors, which include rapid development, the migration of residents from other districts, and the economic conditions in the Langat Basin. There is an average of 22 industrial categories operating around the Langat Basin alone, with electrical and electronic industries dominating the area, followed by manufacturing industries, with at least 337 factories actively operating in this basin.

The geology of the Langat Basin consists of the oldest known rocks (Middle Cambrian or earliest Paleozoic), which include granite, alluvium, sedimentary, and volcanic rocks [31]; the alluvium was found to be consisting of sand, clay, gravel, and silt. The Langat mountainous areas consist of Permian igneous rock and pre-Devonian schist and phyllite [32]. Meanwhile, the lowland part of the Langat Basin is formed from unconsolidated gravel, sand silt, and clay formations. The formation of Kenny Hill and the Kajang formation was build up from sandstone, phyllite, shale, and quartzite [7]. The study area’s aquifer system is made up of alluvial sand, silt, and gravel deposits that form a shallow, confined aquifer, with the unsaturated zone of the aquifer consisting of clay with a thickness ranging between 1 and 3 m [33]. Most of the aquifer ranges from 5 to 80 m in depth. Table 2 shows the geological profile setting of the Langat Basin. The groundwater of the Langat Basin is replenished from the rainfall and river water by (1) downward flow through the aquitard, (2) the intrusion of water along the surfaces of geological outcrops where the aquitard is shallow or unavailable, (3) intrusion from comparatively more porous bedrock, (4) penetration from river bottoms in areas where the river floor is in contact with more porous sand horizons, and (5) intrusion from wetlands and brackish water where the higher aquifer and the aquitard have been removed and replaced by more permeable materials [34].

**Figure 1:**
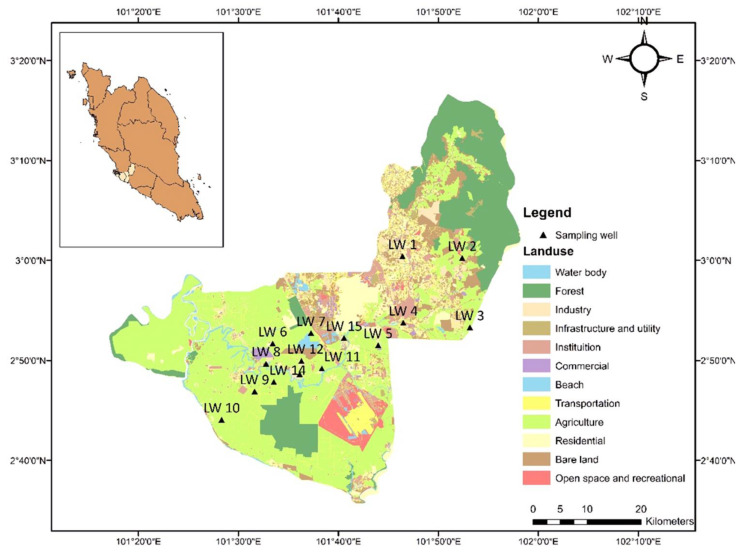
The land use map of the Langat basin.

The Langat Basin has a tropical climate with humid, hot, and wet season conditions throughout the year. The basin is influenced by two types of monsoons, namely the northeast (November–March) and the southwest (May–September) [35]. It receives an annual rainfall ranging from 1500 to 2900 mm (Table 3). The average temperature throughout the year is 27 °C, with high temperatures expected around April and May, while low temperatures occur in November and December. Forty-five groundwater samples were collected from 15 different sampling stations distributed evenly across the Langat basin. The land use of the studied area is approximately comprised of landfill, plantation, residential, and coastal areas. This study was conducted in July 2018. Most of the sampling stations were located in areas that practice industrial and agricultural activities (Table 4). Figure 1 shows the location and the distribution of groundwater sampling stations in the Langat Basin.

### 2.2. Physicochemical Parameter, Major Ions, and Heavy Metals

The sampling locations were determined during the site reconnaissance survey with a sampling design that focused on spatial variations. A total of 45 groundwater samples were collected from 15 boreholes labeled LW1 to LW15 (Figure 1). The groundwater samples were pumped out for about 10–15 min to eliminate stagnant water that could interfere with the analysis of the experiment. During the sampling activities, the in situ data measurements were assessed to obtain a representative measure of the quality of the water and to prevent any possibility of biochemical changes occurring in the samples. In this research study, physicochemical parameters were obtained with triplicate measurement, and the studied parameters involved temperature (Temp, °C), electrical conductivity (EC, µS/cm), total dissolved solids (TDS, mg/L), salinity (ppt), pH level, and dissolved oxygen (DO, mg/L). In situ analyses were measured using a multi-parameter probe YSI 556. All of the probes were calibrated with buffer solutions before and after the sampling to ensure that they functioned accurately and to prevent erroneous measurements from occurring. For major anion analysis, the groundwater samples collected were un-acidified and non-filtered with the exception of sulfate (SO_4_^2−^), which required filtered samples to determine its concentration. The filtered groundwater samples had SulfaVer 4 reagent powder pillow added, which formed a white precipitate with the presence of SO_4_. The amount of precipitate formed was proportional to the concentration of the SO_4,_ which was detected using a HACH spectrophotometer (DR/2500 HACH Odyssey, Loveland, CO, USA). Bicarbonate (HCO_3_) and chloride (Cl) were measured by the titration method according to the standard American Public Health Association method [36].

Concerning the major cations and heavy metals, the groundwater samples were filtered using a 0.45 µm millipore filter (Whatman Milipores, Clifton, NJ, USA) and acidified with nitric acid before being transported to the laboratory of the Faculty of Forestry and Environment, Universiti Putra Malaysia for further analysis. The acidification process was carried out to reduce any chance of precipitation, adsorption, or bacterial activities that would affect the outcome of the results. Groundwater samples were stored and kept at 4 °C to minimize and reduce potential microbial activity in the water samples [37]. The studied heavy metals were iron (Fe), manganese (Mn), arsenic (As), copper (Cu), lead (Pb), zinc (Zn), cadmium (Cd), chromium (Cr), mercury (Hg), nickel (Ni), and selenium (Se). Major ions and heavy metals were analyzed using inductively coupled plasma mass spectrometry (ICP-MS, ELAN DRC-e, Perkin Elmer, Shelton, CT, USA). A variety of standard solutions were freshly prepared using stock standard solutions diluted (ICP Multi-Element Mixed Standard III, Perkin Elmer) with Milli-Q water.

### 2.3. Data Analysis

#### 2.3.1. Statistical Analysis

The statistical data analysis was carried out using the application of IBM SPSS Statistic 23 software version (International Business Machines, New York, USA) to perform univariate analysis and multivariate statistical techniques such as correlation analysis, principal component analysis (PCA), and hierarchical cluster analysis (HCA). The collected data were subjected to descriptive statistics analysis present by its mean and standard deviation. Correlation coefficient analysis was performed to study the association of the parameters with each other [38]. PCA and HCA are reported to be helpful supplemental tools for identifying similarities among geochemical behaviors or common sources for chemicals in groundwater [39,40,41]. Each sampling station was attributed to a large number of physicochemical variables, which make the regional hydrogeochemical study a multivariate problem [42]. Additionally, it was used for evaluating statistical data and identifying the source of the pollutants. There are several methods for determining the limit of detection (LOD) in the analysis. The most commonly used is treating the LOD value as the absolute value (for example, if the limitation was <0.5, then the value of 0.5 would be used); secondly, the researchers would treat the LOD values as zero; third, the LOD values would be excluded from the data set; and finally, the LOD value would be substituted with a value between zero and the LOD value (new average value) [43]. For the current study, this practice was the first choice, but fortunately, all parameters for each station had solid concentration values (Tables S1–S3).

#### 2.3.2. Hydrochemical Facies

Hydrochemical facies of the groundwater can be better explained through the plotting of major cations and anions using a piper trilinear diagram from the application of GW Chart software 1.30.0 software [44,45,46]. It explains the variation of the dominance of anion and cation concentrations. It is also one of the graphical methods that are most commonly used in groundwater chemistry studies [47].

#### 2.3.3. Hierarchical Analysis Component (HCA)

An HCA produces classes that group the groundwater sampling locations or studied quality parameters for enabling a better interpretation of the origin of groundwater contamination [48]. Ward’s method is one of the most common methods used in applying the HCA. The interpretation can be made through the visual inspection of the dendrogram produced by the HCA [49]. The application of an HCA has been widely used in hydrogeochemical studies by grouping the studied parameters based on their similarities [50].

#### 2.3.4. Principal Component Analysis (PCA)

Principle component analysis (PCA) is a powerful multivariate statistical technique used for reducing a large number of variables into smaller components to help interpret data quickly [51]. It provides important information for the whole data set while maintaining the relationships in the original data. The varimax method was applied to execute the rotation of the PCA, and where the PCs had an eigenvalue greater than 1.00, they were retained and discussed. The strength of the physicochemical parameter loading is classified as ‘strong’ (>0.75), ‘moderate’ (0.75 to 0.50), or ‘weak’ (0.50 to 0.30) [52].

## 3. Results and Discussion

### 3.1. Descriptive Analyses

#### 3.1.1. Physicochemical Properties of the Groundwater in the Langat Basin

The descriptive statistics of the hydrogeochemistry of the Langat Basin are outlined in Table 5. Most of the variables range widely, thereby specifying the complexity of the hydrogeochemical conditions occurring in the groundwater. The pH values varied from 4.40 to 7.42 (Table S1), indicating a range between acidic to basic conditions of the groundwater. This condition may be attributed to the application of fertilizer in the surrounding plantation areas, which seeps into the groundwater. Additionally, the low pH value in the study area, showing acidic conditions, is most probably caused by the close distance to the recharge area and, in part, by saltwater intrusion [53,54]. Moreover, the occurrence of natural processes such as water flowing through non-carbonate rock leads towards the range of the recorded pH values. Aside from the presence of an alluvial aquifer in the basin, 73% of the Langat Basin is comprised of the acid intrusive geological unit, which may contribute to the following event [55]. Besides, the experience of an unpleasant odor of rotten eggs, or H_2_S, in some of the sampling stations could be the chemical reaction of the product of SO_4_ reduction where pH values fluctuate. H_2_S describes the anaerobic process in the groundwater that produces the unpleasant odor and could responsible for the pH fluctuation and also subject to the SO_4_ concentration in the groundwater. The groundwater temperature ranged between 28.10 °C and 31.10 °C.

Meanwhile, the DO values ranged from 0.70 mg/L to 2.19 mg/L. The depletion of DO values in the groundwater was influenced by the intrusion or existence of waste discharges or natural organic matter into the groundwater system. The Langat Basin is predominantly occupied by industrial activities dominated by manufacturing activities and plantation activities, and this may have led to the discharge of contaminants from these activities into the groundwater system [56,57]. Furthermore, agricultural activities make up 60% of the total area of the Langat Basin, with many types of pesticides being applied to optimize agricultural production [8]. Thus, the stations in close proximity to these areas have a high tendency to be exposed to the pollution produced by these sources, thus exhibiting low DO values. LW10 experiences high salinity, TDS, and EC values as the location is near to coastal and agricultural activities (Table S1). This has exposed the area to saltwater intrusion and fertilizer seepage [58,59].

#### 3.1.2. Major Ions Distribution in the Langat Basin

The abundance of major ions was in the following order: HCO^3−^ > Cl^−^ > Na^+^ > SO_4_^2−^ > Ca^2+^ > K^+^ > Mg^2+^ (Table S2). The concentration of HCO^3−^ might have been influenced by mineral dissolution such as water–rock interaction, carbonate minerals, or weathering processes that occur in the groundwater [60]. The Cl^−^ concentration for the present study ranged between 19.99 mg/L and 499.85 mg/L, while SO_4_^2−^ readings ranged between 0.07 mg/L and 244 mg/L, with all the water samples found containing SO_4_^2−^ is below the set limit of 250 mg/L [61,62]. SO_4_^2−^ naturally exists in most of the freshwater, but its presence may originate from the application of ammonia sulphate fertilizers in agricultural activities as the basin is dense with agricultural activities. Cl^−^ ions are one of the most important inorganic anions in the groundwater, and may present in the groundwater systems through wastewater, chemical fertilizers, irrigation, or industrial effluents [52]. Since some of the sampling points have high Cl^−^ values, the elevated levels of Cl^−^ will lead to problems, including an increase in water corrosivity and taste interference [5].

Mg^2+^ concentration was found to be between 0.21 mg/L and 25.85 mg/L, and none of the groundwater samples exceeded the limit of 100 mg/L [62]. Mg^2+^ could be originated from carbonate minerals or weathering and leaching processes. For Na^+^, the average threshold is 200 mg/L. High levels of Na^+^ in the groundwater are related to the aquifer recharge from lateral limestone formations or clays by cation exchanges or silicate weathering [63]. The domination of cations found in the studied area shows that the sources are silicate weathering and agricultural runoff [64,65]. The concentration of Ca^2+^ ranged between 0.05 mg/L and 82.65 mg/L. It is commonly found as part of the alkaline earth metal and rock mineral components or from calcium carbonate (CaCO_3_) and dolomite (CaMg(CO_3_)) [66]. The current study indicates that Ca^2+^ is introduced to the groundwater through the weathering of silicate and limestone alterations. The lithology of the Langat Basin comprises six geological units, including limestone [55]. The K^+^ value varied between 0.92 mg/L and 40.51 mg/L. A higher concentration of K^+^ at certain stations indicates that it was derived from the effluent coming from either industrial activities or fertilizer application from the agricultural areas [40].

#### 3.1.3. Occurrences and Distribution of Heavy Metals in the Groundwater of the Langat Basin

The order of heavy metal dominance is as follows: Fe > Mn > Zn > As > Hg > Pb > Ni > Cu > Cd > Se > Cr (Table S3). All the groundwater samples were found to be within the limit set by the Ministry of Health Malaysia [62] and the World Health Organization [61], except for Hg, Mn, and Fe (Table 5). The analysis of the spatial distribution of Hg indicates that the highest concentration was in the areas of residential, coastal, and agricultural practices. Considering that the geology of the Langat Basin consists of pre-Cambrian rock, Hg may originate naturally in the groundwater, but at low concentrations. The Langat Basin has a high amount of industrial activities, which may lead to the atmospheric emission of Hg, which later accumulating in the soil and water [67]. This can be worsened by the wind flow and direction; Malaysia in particular is highly influenced by the tropical monsoon season [68]. The concentrations of Hg in the Langat Basin groundwater are comparatively higher than the maximum Hg detected in other regions such as the Isonzo River Alluvial, Italy (0.0008 mg/L) and Revdalin, Norway (0.0001 mg/L) [69,70]. This can be explained by the urban density development and the site area’s historical land use. The Pearl River Delta, similar to the Langat Basin, was influenced by industrialization in urban and peri-urban areas, with elevated Hg concentrations three times higher than the Hg concentrations in rural areas [39]. However, studies focusing on the occurrences of Hg in urban areas are still scarce, and more attention in future studies is needed for a better understanding regarding the sources, transportation, and fate in groundwater of Hg.

The variation of heavy metal concentrations between stations is probably attributed to the distance factor. The groundwater systems that experienced an increase in contaminants were located closer to pollution sources such as agricultural activities and industrial activities. Fe shows the highest abundance among other types of heavy metals, due to it being among the abundant elements making up the earth’s crust and also being released through intermixed processes such as water–rock interaction and anthropogenic activities [71]. The increased Fe intensity in groundwater is linked to natural origins such as chemical processes in rocks and biological systems. Meanwhile, Mn is considered among the “macro-elements”, which indicates that these elements were by ‘land-based’ discharge, which leads to its release into the groundwater [72]. Other than that, some of the sampling stations were located near industry, urban, and agricultural areas. Anthropogenic activities, such as piping services for industrial and domestic uses, are other potential sources that could have led to these variations in concentration. This includes metals, in particular Mn, which are used in the production of steel. Moreover, metals are widely used in industry, and the corrosion from underground steel pipelines also transfers these elements into the aquifer [54]. The other heavy metals may originate naturally due to geological features or through anthropogenic activities such as direct leachate percolation from landfills and industrial waste dumping [47].

### 3.2. Correlation Matrix

The correlation coefficient between physicochemical parameters was evaluated to determine any significant variations between the variables (Table 6). A robust positive correlation coefficient was found between TDS-Sal, TDS-EC, and Sal-EC (*r* = 1.000, *p* < 0.01). EC measures the ability of the water to transmit electric current by dissolved ions. Meanwhile, TDS is described as the sum of ions, cations, and anions in the water. Hence, TDS could indicate the behavior of the EC of the water [73]. It is also used to reflect the salinity of the groundwater. Therefore, salinity, TDS, and EC are correlated to each other, in which the increase of one gives rise to another. HCO_3_ also shows a moderate positive correlation with TDS, EC, and salinity *r* = 0.625 to 0.641 (*p* < 0.01). The moderate correlation between TDS, EC, and salinity and HCO_3_^−^ suggests that this type of ion mostly controls these parameters. HCO_3_^−^ shows a strong correlation with Cl^−^ (*r* = 0.761, *p* < 0.01). Thus, it suggests that these ions may come from the same source, which might indicate the chemical characteristics of a meteoric water mix, with water coming from anthropogenic activities [74]. 

A positive correlation was found between SO_4_ with Cl^−^ (*r* = 0.544) at a significant level *p* < 0.01. A moderate relationship between these major salinity components, SO_4_^2−^ with C^−^, indicates that it either occurs due to saltwater intrusion in the groundwater system or is discharged from industrial and agricultural activities [73]. Na, K, Mg, and Ca show a positive and significant correlation with each other (*r* = 0.689 to 0.981) at a significant level *p* < 0.01. The anions may reflect the occurrence of silicate weathering in the groundwater system, as this process can release major cations into the groundwater [75]. It was also found that these ions might present in the groundwater through anthropogenic brine [47]. A positive correlation (*r* = 0.981, *p* < 0.01) of Mg-K may signify the leaching of salts from secondary salts [66]. Na-K strong positive correlation (*r* = 0.930, *p* < 0.01) signifies that these ions have a common origin, where the presence of these ions may come from clay [76]. Mg-Na correlation (*r* = 0.915, *p* < 0.01) reflects the occurrence of cation exchange between Mg and Na occurring in the groundwater system.

The Pearson correlation performed for heavy metals with physicochemical parameters shows that they have a moderate correlation with each other. This may reflect that these elements did not originate from single sources [77]. Instead, the presence of these heavy metals may be due to various anthropogenic processes and geogenic processes [78]. Some of the metals show a strong positive relationship with other elements and parameters. Mn was found to have strong positive correlation with TDS (*r* = 0.820, *p* < 0.01), salinity (*r* = 0.832, *p* < 0.01), and EC (*r* = 0.827, *p* < 0.01). This may reflect that these parameters were influenced by the concentration of Mn. Se and Fe also showed a moderate correlation with some of the studied parameters (Table 6). The varied correlation between heavy metals and pH is related to the mobility of the heavy metals. This is dependent on the psychochemical parameters, where the solubility of the heavy metals increased with the decrease of pH values, which clarifies that the correlation occurs between heavy metals and physicochemical parameters. Fe is highly correlated with Mn, which suggests that they might come from a common source [79]. Fe and Mn can be released from geogenic sources as they are common elements in rocks and minerals [80,81]. Cd showed a moderate correlation with Zn (*r* = 0.549, *p* < 0.01) and Pb (*r* = 0.548, *p* < 0.01). These demonstrate that these heavy metals may originate from the same industrial activities, such as through leachate release [81].

### 3.3. Hydrochemical Facies

**Figure 2:**
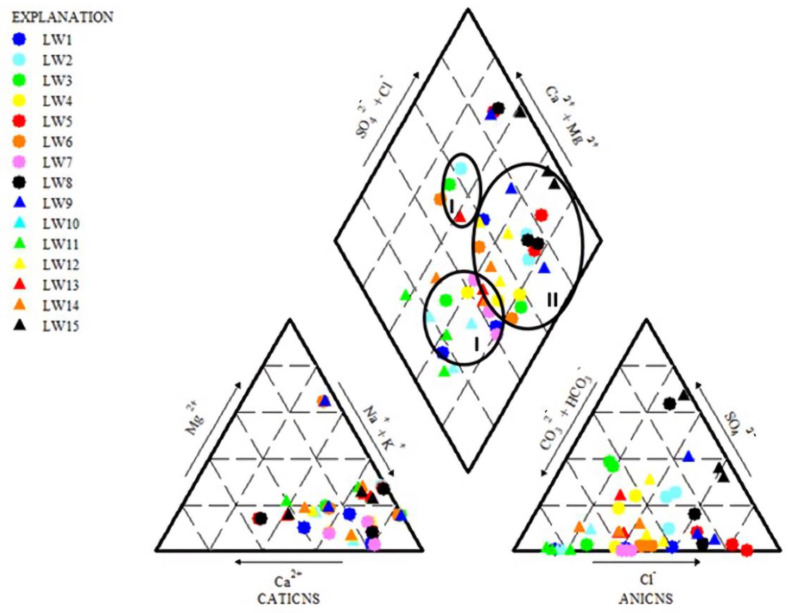
Piper trilinear diagram showing hydrogeochemical facies. Na-HCO_3_ water type (Circle I) and Na-Cl water type (circle II).

The hydrochemical facies of the groundwater samples were Na-HCO_3_ (Circle I) and Na-Cl (Circle II) water types (Figure 2). A Na-HCO_3_ water type suggests that there is an occurrence of water–rock interaction in the groundwater system [82]. Na-HCO_3_ presence is due to the cation exchange process with clay and clay minerals. Generally, it shows mix-water type characteristics [83]. Meanwhile, the Na-Cl water type that dominates the groundwater indicates a saline water type. The source for this water type is likely due to the salt deposits from agricultural activities that flow into the groundwater [55]. However, this also suggests that it is probably governed by silicate weathering and mineral dissolution [80]. Na^+^ and Cl^−^ may also originate from clay materials, which may introduce these ions into the groundwater system, hence influencing the water type [84]. The piper plot reveals that the groundwater chemistry of the Langat Basin belongs to the Na-HCO_3_-Cl water type. This mixing is also directed to the freshwater mixed with saline that is influenced by agricultural, urban, and industrial activities that contribute to the salinization of the groundwater.

**Figure 3:**
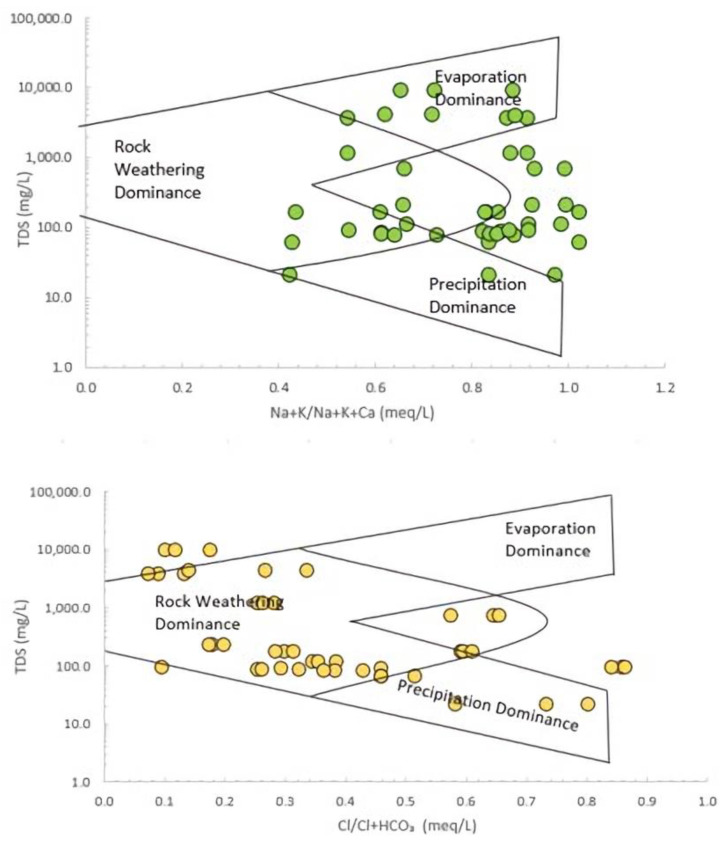
The Gibbs diagram showing the mechanism governing groundwater chemistry.

The Gibbs diagram (Microsoft Corporation, Washington, USA) [85] is an important tool for evaluating the functional source of dissolved ions in water such as the dominance of precipitation, rock weathering, and evaporation dominance that govern the water chemistry. From the plots, it is evident that the groundwater chemistry of the Langat Basin has not been regulated by any dominant mechanism (Figure 3). The Gibbs diagram plot revealed that anions of the groundwater samples had been driven by rock weathering processes. The presence of HCO_3_^−^ in the groundwater occurs due to the dissolution of rock weathering processes [60]. It was also revealed that precipitation factors and anthropogenic activities have also played a role in the cation concentration in the groundwater. Anthropogenic activities may have introduced not only HCO_3_^−^ but also Cl^−^ into the groundwater of the Langat Basin through the precipitation process, consequently leading to elevated concentrations of both anions in the groundwater, especially in the wells labeled LW10, LW11, LW13, and LW14. It is also evident that the ionic mechanism of cations in the groundwater of the Langat Basin appears to be influenced mostly by rock-weathering and anthropogenic processes. The groundwater samples that fell outside of the diagram are suggested to have been influenced by anthropogenic mechanisms [58]. Despite the inclusion of anthropogenic processes, the cations in the groundwater system are still within the limits.

### 3.4. Principal Component Analysis (PCA)

**Figure 4:**
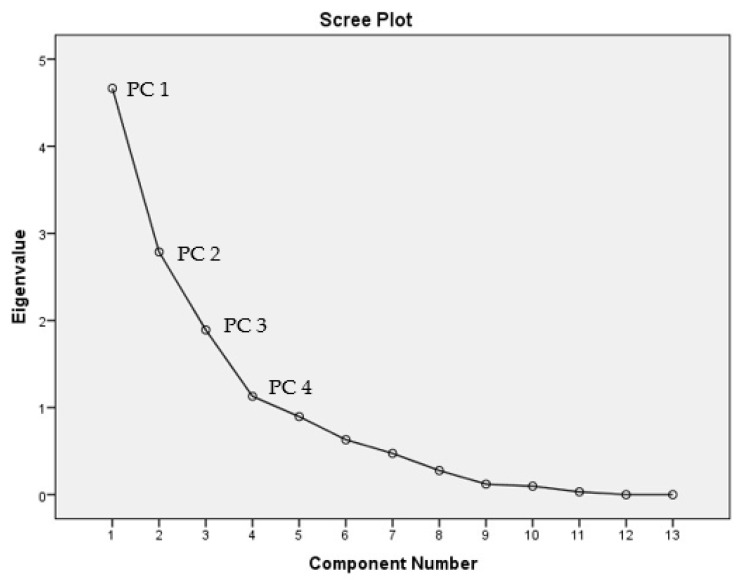
Scree plot for PCA.

**Figure 5:**
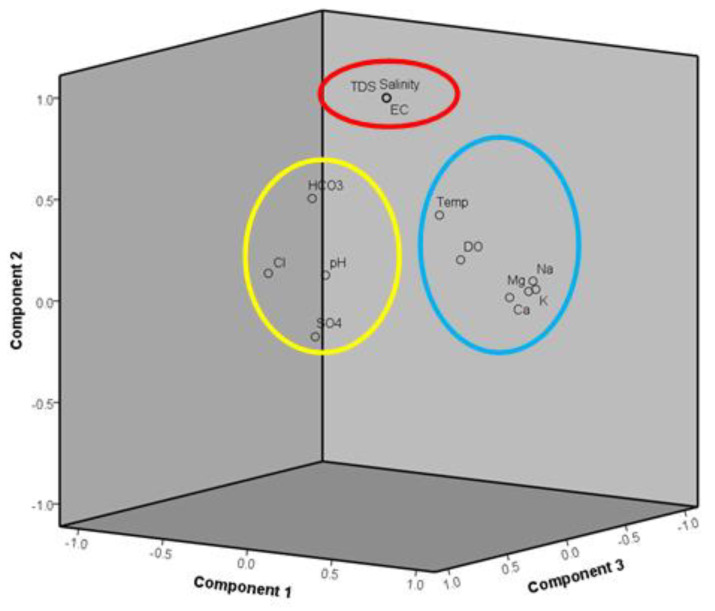
Rotated component of PCA.

PCA helps to determine the most significant factors that account for the significant patterns [86]. The Kaiser–Meyer–Olkin (KMO) test values of 0.557 and Barlett’s test of sphericity (*p* < 0.001) showed that the data were sufficient for analysis using PCA [87]. PCA produced four major components with eigenvalues, explaining 81% of the variance in the data set (Figure 4). PC1 explained 26.97% of the total variance, with a strong positive loading found for all major cations, which were K^+^, Mg^2+^, Na^+^, and Ca^2+^. The loading of the major cations indicates that the weathering of rock minerals occurred in the groundwater [26]. Cations are commonly presented in clay minerals and become a source for groundwater through silicate weathering or carbonate weathering. Nevertheless, this may also be attributed to the anthropogenic activities adjacent to the land use [50]. PC2 accounts for 26.83%, with the loading showing a strong positive loading between TDS, salinity, and EC. This represents that the same factor might influence these parameters. Based on the findings, these parameters show a moderate correlation with HCO_3_, and this suggests that the parameters are directly affected by this ion (Table 7) (Figure 5).

PC3 exhibits a moderate to strong positive loading of major anions (HCO_3_^−^, SO_4_^2−^ and Cl^−^) with a total variance of 17.89% The interaction between water, soils, and rock and the weathering of silicate might result in releasing major anions into the groundwater [56,88]. The presence of this ion may be due to anthropogenic activities that introduce these ions into the water body, mostly through the application of chemical fertilizers in agricultural activities [89,90]. The presence of HCO_3_^−^ can be due to the decaying of soil organic matter incorporated with the dissolving of CO_2,_ reflecting the influence of fertilizer [91]. PC1 to PC3 might imply that these loadings are related to anthropogenic and natural occurrences. PC4 explains 8.88% of the total variance and is characterized by the moderate loading of pH and DO and the negative loading of temperature. This represents that the high value for DO and pH is more likely at low-temperature values. For the pH level, ionization might occur where the H^+^ in solution will decrease with temperature and increase the pH level. As for the inverse relationship between DO and temperature, this is due to the solubility of oxygen in the water.

### 3.5. Hierarchical Component Analysis (HCA)

**Figure 6:**
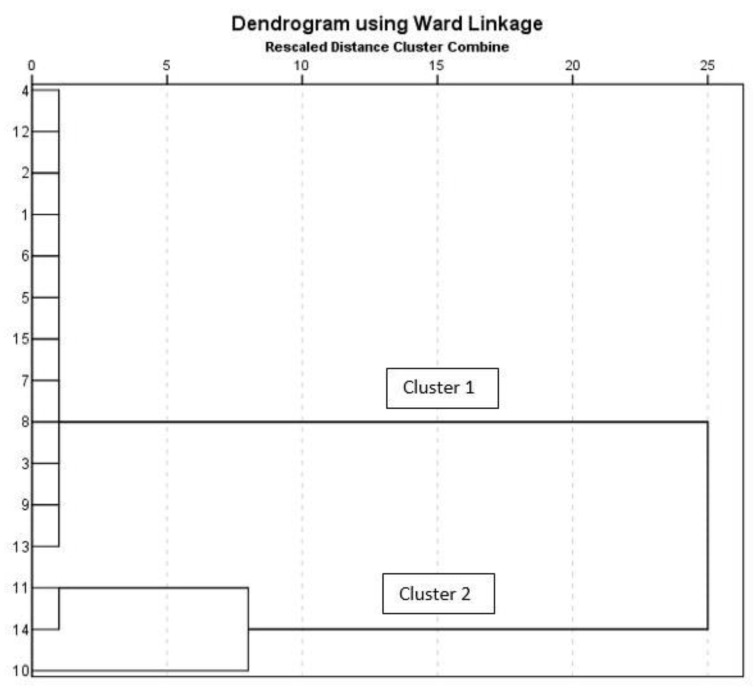
Classification of the sampling stations based on HCA outputs.

HCA classified the sampling stations into two major clusters, with 80% of the stations belong to cluster 1 and the other stations being classified under cluster 2 (Figure 6). Cluster 1 consists of stations LW1 to LW9, LW12, LW13, and LW15. In this cluster group, the sampling stations had lower pollution levels. It was found that the stations belonging to this cluster were located far from pollution sources such as industrial discharge, construction projects, or farming activities. These sampling stations were therefore less exposed to groundwater pollution, which is supported by the results obtained from the present study, which show that almost all of the stations in this cluster did not exceed any permissible limit established by the World Health Organization (WHO) and Ministry of Health Malaysia (MOH).

Cluster 2 consists of the sampling stations LW10, LW11, and LW14; these stations have been recognized as having a mix-water type. LW10 was located near the coastal area, whereas stations LW11 and LW14 were located in an area that was close to a palm oil plantation and a residential area. Station LW11 was also located within an area with active farming activities, and a construction site was also located nearby to station LW14. Station LW14 had the highest Cl value, which might be attributed to the nearby agricultural activities (Table S2) (Figure S1). The Langat Basin comprises the alluvial aquifer, and the groundwater recharge in the alluvial aquifer occurs via a few mechanisms, such as the direct infiltration of rainfall and the return flow of irrigated water [92]. These mechanisms will introduce pollutants to the groundwater system through seepage.

## 4. Conclusions

The hydrogeochemical characteristics and the processes affecting the chemistry of the groundwater of the Langat Basin have been evaluated based on the physicochemical parameters and the major ions. The current study revealed that the dominant cations were Na^+^ > K^+^ > Mg^2+^ > Ca^2+^, while the dominant anions were HCO_3_^−^ > Cl^−^ > SO_4_^2−^. The water types found in the groundwater system were Na-HCO_3_, Na-Cl, and Na-HCO_3_-Cl. The TDS and EC in certain wells (stations LW9, LW10, LW11, LW13, and LW14) exceeded permissible limits and showed high values reflecting that the groundwater was unfit to use without proper treatment and management. The dominance of heavy metals was as follows: Fe > Mn > Zn > As > Hg > Pb > Ni > Cu > Cd > Se > Sr. For the studied heavy metals, only Hg was found to exceed the permissible limits. Natural and anthropogenic activities, specifically industrial and agricultural activities, have demonstrated an effect and dominant influence over the Langat Basin’s groundwater chemistry. Most of the sampling stations with contaminated groundwater were located near pollution sources.

Considering the intense industrialization and urbanization in the Langat Basin, it is crucial to have continuous monitoring and risk assessments to prevent future deterioration of the groundwater and to develop practical groundwater management. The findings of the study are decisive in evaluating the current quality of the groundwater and the sources of contamination of the groundwater system. Despite most of the parameters being below the permissible limits, the contaminants found were significant. The results could help to design more efficient land development to reduce groundwater contamination. Furthermore, the multivariate analysis approach is important for understanding the environmental condition, allowing for the evaluation of the ecological risk of groundwater, and assisting in identifying the priorities for sustainable groundwater management, specifically in the tropical basins in other parts of the region. Future groundwater quality studies for carrying out seasonal sampling in the Langat Basin are recommended.

## Figures and Tables

**Table 1:** Summary of inland groundwater contamination studies in Malaysia.

Study Area	Method	Studied Elements	Reference
North of Kuala Lumpur, Kinta Valley, Perak and Alor Setar, Kedah	Hydrochemistry analysis	Major ions	[12]
Disposal site in Seri Petaling, Selangor	2D electrical resistivity, geochemistry, and Analysis of variance (ANOVA)	Resistivity image, in situ parameters, major ions and trace elements	[13]
Disposal site in Taiping Perak	Hydrochemistry analysis, surfer software	Soil samples, heavy metals	[14]
Langat Basin, Selangor	2D resistivity measurement, MODFLOW (modular finite-difference flow model), and SWOT (Strengths, Weaknesses, Opportunities, and Threats) analysis	In situ parameters, major ions, and heavy metals	[8]
Ampar Tenang landfill, Selangor	Sodium adsorption ratio (SAR), piper diagram, 2D resistivity technique	Resistivity image and major ions	[15]
Rosob Village, Sabah	Hydrochemistry analysis	Heavy metals	[16]
Ampar Tenang and Bukit Tagar landfill, Selangor	Hydrochemistry analysis	Major ions, trace elements, and heavy metals	[17]
North Kelantan	2D resistivity measurement, hydrochemistry, and soil particle size analysis	Resistivity image, soil samples, major ions	[18]
Ampar Tenang landfill, Selangor	Physiochemical and biochemical analysis	Physiochemical parameters, organic contaminants, and heavy metals	[19]
North of Gemas, Negeri Sembilan	2D electrical resistivity measurement, induced polarization, and borehole geophysical techniques	Resistivity images, pumping test data, and rock and soil data	[20]
Melaka state	DRASTIC model (Depth to water, Recharge, Aquifer media, Soil media, Topography, Impact of the vadose zone, Hydraulic conductivity) with GIS and remote sensing	Groundwater level, recharge, lithology, soil type, topography, hydraulic conductivity, and land use data	[21]
Southwest state of Selangor within the Langat Basin	Bank infiltration (BI), pumping test, 2D resistivity survey, soil sieve analyses	Resistivity images, isotope and major ions	[22]
Batang Padang, Perak	GIS-based optimized DRASTIC model and analytic Process (AHP)	Borehole data, average annual rainfall, geophysical data, soil map, remote sensing imagery, and geophysical data	[23]
Ex-landfill Taman Beringin, Selangor	Hydrochemical analysis, physicochemical analysis, and water quality analysis	Physicochemical parameters, heavy metals, NH_3_-N, Cl, F, Pb, Ni, and Fe	[24]
Semenyih and Kajang, Selangor	Geological analysis, hydrochemical analysis, piper diagram, and well water chemistry interpretation	Major ions, physicochemical parameters,	[25]
Langat Basin	Hydrochemical analysis, piper diagram, multivariate analysis	Physicochemical parameters, major ions, and heavy metals	Current study

**Table 2:** The geological profile setting of the Langat Basin [34].

A.s.l (m)	Geological Setting
>30	acid intrusives (undifferentiated)
>20	Phyllite, schist, and slate; limestone and sandstone
>10	Peat, humic clay, and silt; clay, silt, sand, and gravel
>1	Clay and silt

A.s.l, average sea level.

**Table 3:** The description of hydrogeological and hydrochemical properties of the Langat Basin [34].

Hydrology	Mean
Temperature	27 °C
Annual precipitation	2200 mm
Annual evapotranspiration	1284 mm
Groundwater recharge rate	108 mm
Groundwater discharge rate	846 mm
Transmissivity	0.024 m^2^/s
Vertical hydraulic conductivity/permeability	7 × 10^−9^ m/s
Aquifer thickness	10–100 m
Sustainable pumping rate	26,849 million liter per day (MLD)
Water type	Ca-Mg-Cl [15] Na-Cl (current study)

MLD: million liters/day.

**Table 4:** Site description of the study areas.

Stations	A.s.l	Coordinate		Water Table	Depth of Borehole	Site Description
	m	N	E	m	m	
LW1	30	3.00705	101.773	5.35	11.0	Residential area
LW2	47	3.00409	101.873	1.00	NA	Residential area and near to a palm oil plantation
LW3	50	2.88834	101.886	3.50	NA	Residential area
LW4	20	2.89599	101.774	3.12	45.0	Near to a river and a shrub area
LW5	36	2.85851	101.732	2.91	22.7	Residential area
LW6	26	2.86136	101.557	4.96	13.0	Residential area and near to a river
LW7	11	2.87883	101.621	4.23	11.7	Wetland and near to a palm oil plantation
LW8	4	2.82795	101.546	3.42	49.5	Part of agricultural activities
LW9	6	2.78176	101.527	4.58	NA	Near to a l palm oil plantation and a lake, and part of agricultural activities
LW10	5	2.73455	101.472	2.00	65.0	Residential area, near to a coastal area and agricultural activities
LW11	10	2.81993	101.639	3.7	15.8	Residential area, near to a palm oil plantation and farming activities
LW12	8	2.83261	101.605	8.75	16.0	Residential area, and near to industrial activities
LW13	11	2.81055	101.602	8.87	NA	Near to industrial areas and a palm oil plantation
LW14	7	2.79758	101.559	0.83	NA	Residential area, near to a palm oil plantation and a construction site
LW15	11	2.87069	101.676	2.93	18.0	Residential area

A.s.l., average sea level. NA, not available from the database. Borehole screening, 4.00−10.00 m (current study). Clay covering, 1–3 m [33].

**Table 5:** Statistical summary of the physiochemical and heavy metal parameters in the groundwater of the Langat Basin.

Parameters	Unit	Mean (SD)	WHO	MOH
pH		6.13 ± 0.89	6.5–8.5	6.5–9.0
Temperature	°C	29.64 ± 0.90	-	-
DO	mg/L	1.25 ± 0.35	-	-
Salinity	ppt	1.14 ± 2.22	-	-
EC	µS/cm	2286.38 ± 4318.75	500	-
TDS	mg/L	1352.58 ± 2534.34	500	1000
HCO_3_^−^	mg/L	185.93 ± 241.87	500	-
Cl^−^	mg/L	74.64 ± 86.77	250	250
SO_4_^2−^	mg/L	38.6 ± 58.22	250	250
Ca^2+^	mg/L	14.58 ± 22.01	75	-
Mg^2+^	mg/L	6.94 ± 6.74	50	150
K^+^	mg/L	12.85 ± 13.87	-	-
Na^+^	mg/L	24.41 ± 19.64	200	200
Fe	mg/L	6.035 ± 9.027	NA	0.3
Mn	mg/L	0.378 ± 0.600	NA	0.1
As	mg/L	0.01 ± 0.019	0.01	0.01
Cu	mg/L	0.002 ± 0.002	2	1
Pb	mg/L	0.005 ± 0.002	0.01	0.01
Zn	mg/L	0.023 ± 0.024	NA	3
Ni	mg/L	0.003 ± 0.001	0.07	0.02
Cd	mg/L	0.001 ± 0.000	0.003	0.003
Se	mg/L	0.001 ± 0.001	0.04	0.01
Cr	mg/L	0.001 ± 0.002	0.05	0.05
Hg	mg/L	0.010 ± 0.013	0.0006	0.001

SD, standard deviation; WHO, World Health Organization; MOH, Ministry of Health Malaysia. NA, not available.

**Table 6:** Pearson’s product moment correlation of the physicochemical parameters and major ions.

	pH	Temp	Sal	Cond	DO	TDS	HCO_3_^−^	Cl^−^	Ca^2+^	Mg^2+^	K^+^	Na^+^	SO_4_^2−^
pH	1	−0.055	0.191	0.201	0.127	0.206	0.429 **	0.398 **	0.013	0.041	0.052	0.103	0.261
Temp		1	0.306 *	0.307 *	−0.010	0.302 *	−0.047	−0.061	0.115	0.235	0.210	0.199	0.056
Sal			1	1.000 **	0.100	1.000 **	0.625 **	0.266	0.179	0.256	0.218	0.244	0.018
Cond				1	0.111	1.000 **	0.631 **	0.272	0.184	0.265	0.228	0.253	0.021
DO					1	0.110	−0.155	−0.213	0.074	0.235	0.205	0.149	−0.126
TDS						1	0.641 **	0.281	0.186	0.269	0.232	0.256	0.026
HCO_3_^−^							1	0.761 **	0.161	0.216	0.229	0.224	0.342 *
Cl^−^								1	−0.026	0.004	0.032	0.048	0.544 **
Ca^2+^									1	0.688 **	0.692 **	0.689 **	0.165
Mg^2+^										1	0.981 **	0.915 **	0.097
K^+^											1	0.930 **	0.131
Na^+^												1	0.018
SO_4_^2−^													1

** Correlation is significant at the 0.01 level (2-tailed). * Correlation is significant at the 0.05 level (2-tailed).

**Table 7:** Principal component loading of the measured variables.

Parameters	PC1	PC2	PC3	PC4
K^+^	0.958	0.112	0.041	0.024
Mg^+^	0.940	0.105	0.076	0.139
Na^+^	0.929	0.147	0.022	0.026
Ca^+^	0.814	0.060	0.057	−0.091
Salinity	0.111	0.981	0.097	0.011
EC	0.120	0.980	0.102	0.022
TDS	0.123	0.979	0.111	0.026
Cl^−^	−0.037	0.212	0.882	0.038
SO_4_^2−^	0.146	−0.102	0.745	−0.100
HCO_3_^−^	0.105	0.571	0.714	0.096
DO	0.215	0.121	−0.385	0.734
pH level	0.042	0.157	0.510	0.576
Temperature	0.234	0.373	−0.178	−0.482
Eigenvalue	3.505	3.488	2.326	1.154
Total variance	26.965	26.831	17.891	8.880
Total cumulative	26.965	53.796	71.686	80.566

## Data Availability

Not applicable.

## Supplementary Materials

The following are available online at https://www.mdpi.com/article/10.3390/ijerph18115733/s1, Table S1: Descriptive analysis for in-situ parameters., Table S2: Major ions descriptive analysis., Table S3. Heavy metals descriptive analysis. Figure S1. Major ions concentration distribution along with sampling station.
