# Supplementary material for: Spatial Analysis of Groundwater Hydrochemistry through Integrated Multivariate Analysis: A Case Study in the Urbanized Langat Basin, Malaysia
Source: Int J Environ Res Public Health. 2021 May 27;18(11):5733. doi: 10.3390/ijerph18115733 (PMC8198349; doi:10.3390/ijerph18115733)
Supplement: Supplementary file 1 [file ijerph-18-05733-s001.zip › ijerph-1197578-supplementary.pdf]

International Journal of
Environmental Research and Public Health

MDPI

Table S1. Descriptive analysis for in-situ parameters.

Stations		pH	Temperature (°C)	Salinity (ppt)	EC μS/cm	DO (mg/L)	TDS (mg/L)
LW1	Mean	5.82	28.70	0.06	146.43	1.06	88.40
	SD	0.01	0.00	0.00	2.46	0.14	1.95
LW2	Mean	5.14	31.03	0.04	108.40	0.70	63.05
	SD	0.01	0.06	0.00	0.00	0.08	0.00
LW3	Mean	7.35	30.50	0.16	372.67	1.35	219.05
	SD	0.05	0.00	0.00	0.06	0.04	0.00
LW4	Mean	6.03	28.67	0.06	135.57	1.33	82.55
	SD	0.00	0.06	0.00	0.49	0.01	0.00
LW5	Mean	4.61	29.40	0.01	36.10	1.67	21.45
	SD	0.01	0.00	0.00	0.00	0.06	0.00
LW6	Mean	6.12	28.10	0.08	186.20	1.21	114.40
	SD	0.02	0.00	0.00	0.61	0.02	0.00
LW7	Mean	5.70	29.67	0.12	281.83	1.07	169.00
	SD	0.00	0.06	0.00	0.12	0.04	0.00
LW8	Mean	6.16	28.70	0.12	280.73	1.36	170.30
	SD	0.01	0.00	0.00	0.06	0.05	0.00
LW9	Mean	7.42	30.60	0.53	1206.00	1.55	708.50
	SD	0.01	0.00	0.00	0.00	0.02	0.00
LW10	Mean	6.11	30.90	8.31	16106.00	1.22	9397.33
	SD	0.01	0.00	0.00	0.00	0.01	7.11
LW11	Mean	6.48	29.80	3.08	6268.67	2.12	3731.00
	SD	0.08	0.00	0.00	0.58	0.07	0.00
LW12	Mean	6.81	29.30	0.06	135.87	0.86	81.25
	SD	0.02	0.00	0.00	0.06	0.03	0.00
LW13	Mean	6.69	29.90	0.91	1985.67	1.37	1183.00
	SD	0.01	0.00	0.00	0.58	0.03	0.00
LW14	Mean	7.14	28.90	3.48	6888.00	0.83	4166.50
	SD	0.00	0.00	0.01	1.00	0.08	6.50
LW15	Mean	4.40	30.50	0.07	157.50	1.04	92.95
	SD	0.00	0.00	0.00	0.00	0.01	0.00

SD, standard deviation.

Table S2. Major ions descriptive analysis.

Stations		HCO_3^- (mg/L)	Cl^- (mg/L)	SO_4^{2-} (mg/L)	Ca^{2+} (mg/L)	Mg^{2+} (mg/L)	K^+ (mg/L)	Na^+ (mg/L)
LW1	Mean	174.87	48.98	2.00	7.41	1.39	4.93	13.23
	SD	134.03	12.12	0.00	8.29	1.14	1.43	8.780
LW2	Mean	55.31	50.65	27.33	4.70	1.12	1.98	4.98
	SD	5.64	1.16	13.32	6.58	0.68	2.02	2.50
LW3	Mean	139.08	31.66	62.67	6.86	7.27	7.90	18.94
	SD	4.23	3.79	50.81	10.65	3.46	1.98	11.74
LW4	Mean	63.44	24.66	16.00	7.59	1.53	4.05	15.30
	SD	10.64	4.16	13.23	8.20	1.19	1.14	7.79
LW5	Mean	11.39	26.99	1.67	4.67	1.08	1.87	4.70
	SD	5.08	3.00	2.08	6.61	0.72	1.98	2.05
LW6	Mean	66.69	37.99	3.00	7.02	7.33	7.56	19.69
	SD	3.73	2.65	0.00	10.61	3.40	2.15	12.43
LW7	Mean	99.23	42.66	0.00	7.81	1.53	3.95	15.49
	SD	1.41	3.06	0.00	8.40	1.19	1.51	8.19
LW8	Mean	50.43	75.65	92.00	4.65	1.10	1.89	5.12
	SD	2.82	3.22	132.18	6.42	0.70	2.12	2.62
LW9	Mean	84.59	142.95	73.00	6.845	7.35	7.72	19.49
	SD	3.73	20.80	100.48	10.63	3.51	1.88	12.02
LW10	Mean	432.69	66.98	12.67	19.51	6.67	13.44	29.12
	SD	5.08	22.86	28.11	26.58	7.83	17.42	26.63
LW11	Mean	248.88	27.66	0.67	33.35	18.72	35.13	51.33
	SD	2.44	9.29	1.16	41.34	6.17	6.89	7.90
LW12	Mean	51.24	33.32	16.00	20.43	8.56	16.03	31.30
	SD	4.23	4.73	20.81	26.24	7.47	15.69	22.48
LW13	Mean	349.73	128.29	69.00	33.99	15.89	35.51	54.00
	SD	5.64	11.37	56.31	42.15	0.89	7.20	3.49
LW14	Mean	953.23	333.23	129.33	20.18	8.59	15.90	28.49
	SD	25.04	173.85	43.88	25.72	7.39	15.75	17.73
LW15	Mean	8.13	47.99	69.00	33.76	15.99	34.91	54.96
	SD	1.41	3.60	56.31	41.68	1.166	6.861	6.22

SD, standard deviation.

Table S3. Heavy metals descriptive analysis.

Stations		Fe (µg/L)	Mn (µg/L)	As (µg/L)	Cu (µg/L)	Pb (µg/L)	Zn (µg/L)	Ni (µg/L)	Cd (µg/L)	Se (µg/L)	Cr (µg/L)	Hg (µg/L)
1	Mean	7.3067	0.1660	0.0211	0.0010	0.0029	0.0160	0.0019	0.0002	0.0004	0.0002	0.0163
	SD	0.1686	0.0046	0.0003	0.0003	0.0010	0.0027	0.0000	0.0000	0.0002	0.0000	0.0241
2	Mean	0.0593	0.0833	0.0012	0.0017	0.0051	0.0223	0.0041	0.0003	0.0011	0.0062	0.0257
	SD	0.0070	0.0014	0.0005	0.0001	0.0015	0.0060	0.0002	0.0002	0.0005	0.0027	0.0107
3	Mean	0.0952	0.1748	0.0002	0.0002	0.0035	0.0133	0.0032	0.0002	0.0005	0.0009	0.0100
	SD	0.0023	0.0024	0.0000	0.0000	0.0004	0.0006	0.0005	0.0000	0.0001	0.0004	0.0045
4	Mean	15.2200	0.6423	0.0631	0.0021	0.0060	0.0265	0.0032	0.0002	0.0005	0.0002	0.0021
	SD	0.2193	0.0069	0.0008	0.0010	0.0002	0.0025	0.0002	0.0000	0.0000	0.0000	0.0025
5	Mean	0.2788	0.1642	0.0002	0.0074	0.0055	0.0371	0.0067	0.0002	0.0006	0.0002	0.0053
	SD	0.0108	0.0034	0.0000	0.0004	0.0026	0.0041	0.0002	0.0000	0.0002	0.0000	0.0039
6	Mean	6.6833	0.5260	0.0499	0.0008	0.0042	0.0140	0.0019	0.0002	0.0005	0.0002	0.0094
	SD	0.1474	0.0036	0.0013	0.0001	0.0013	0.0060	0.0000	0.0001	0.0000	0.0000	0.0038
7	Mean	12.6033	0.2725	0.0002	0.0019	0.0046	0.0156	0.0019	0.0002	0.0010	0.0002	0.0029
	SD	0.0709	0.0009	0.0000	0.0006	0.0005	0.0073	0.0000	0.0000	0.0007	0.0000	0.0006
8	Mean	2.3867	0.0291	0.0051	0.0011	0.0063	0.0185	0.0019	0.0004	0.0009	0.0002	0.0031
	SD	0.0231	0.0004	0.0002	0.0002	0.0049	0.0112	0.0000	0.0004	0.0005	0.0000	0.0024
9	Mean	0.0545	0.0033	0.0002	0.0032	0.0057	0.1001	0.0020	0.0005	0.0009	0.0002	0.0138
	SD	0.0120	0.0004	0.0000	0.0011	0.0016	0.0296	0.0002	0.0003	0.0005	0.0000	0.0073
10	Mean	34.0367	2.4617	0.0072	0.0040	0.0040	0.0080	0.0019	0.0002	0.0018	0.0002	0.0271
	SD	0.7149	0.0510	0.0008	0.0005	0.0010	0.0033	0.0000	0.0001	0.0011	0.0000	0.0319
11	Mean	1.9033	0.3862	0.0002	0.0028	0.0062	0.0109	0.0023	0.0002	0.0007	0.0002	0.0092
	SD	0.0306	0.0554	0.0000	0.0010	0.0016	0.0022	0.0008	0.0000	0.0002	0.0000	0.0067
12	Mean	0.2918	0.0412	0.0013	0.0012	0.0065	0.0205	0.0019	0.0002	0.0008	0.0002	0.0052
	SD	0.0033	0.0053	0.0004	0.0001	0.0015	0.0055	0.0000	0.0000	0.0005	0.0000	0.0038
13	Mean	9.1879	0.5882	0.0002	0.0014	0.0053	0.0147	0.0020	0.0003	0.0010	0.0002	0.0107
	SD	0.3757	0.0222	0.0000	0.0001	0.0014	0.0028	0.0002	0.0001	0.0006	0.0000	0.0144
14	Mean	0.3536	0.1130	0.0004	0.0023	0.0014	0.0009	0.0019	0.0002	0.0012	0.0003	0.0039
	SD	0.0073	0.0079	0.0003	0.0002	0.0003	0.0004	0.0000	0.0000	0.0004	0.0001	0.0026
15	Mean	0.0667	0.0204	0.0002	0.0021	0.0089	0.0263	0.0019	0.0003	0.0005	0.0002	0.0006
	SD	0.0057	0.0005	0.0000	0.0002	0.0031	0.0028	0.0000	0.0001	0.0000	0.0000	0.0003

SD, standard deviation.

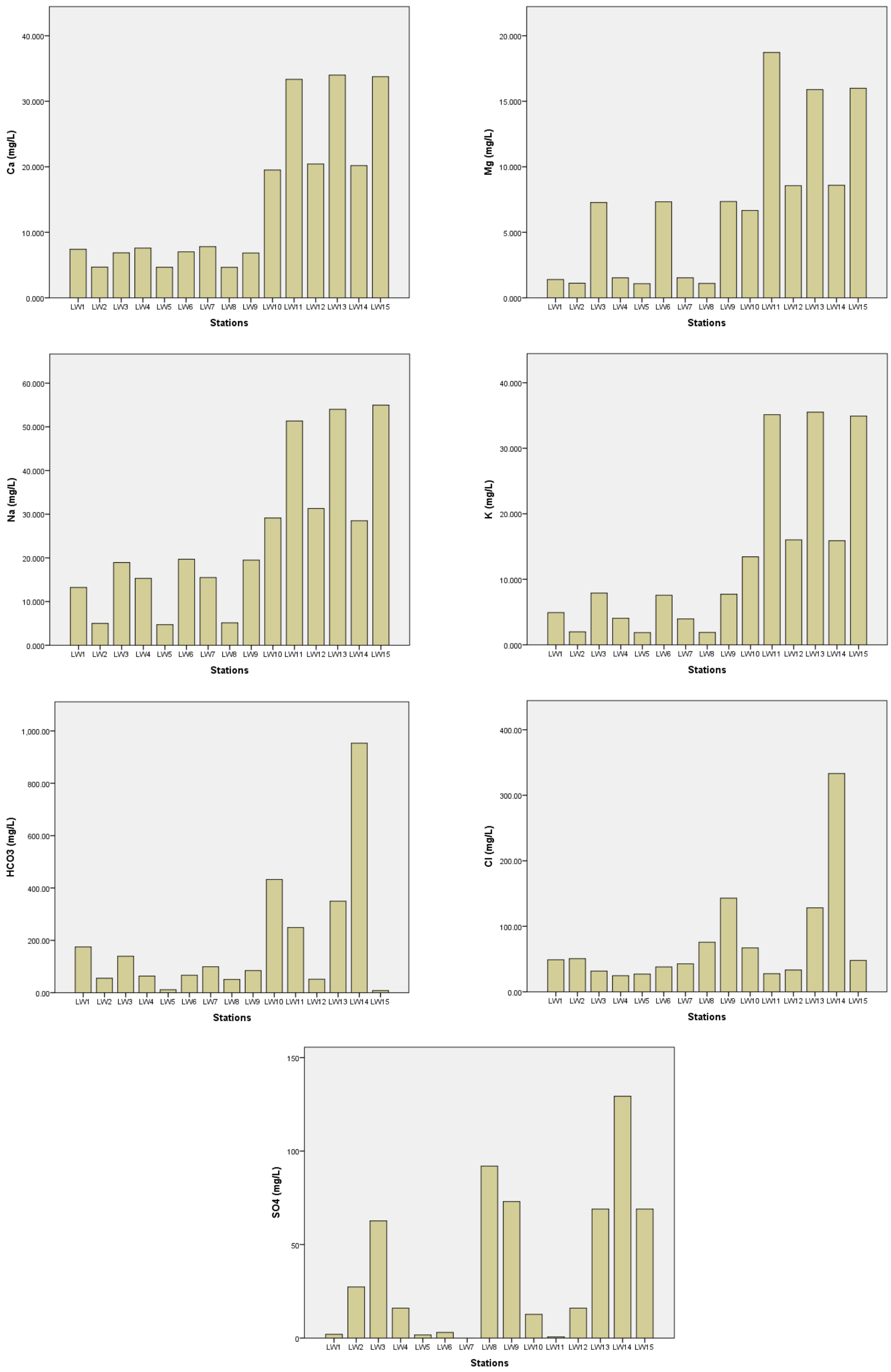

Figure S1. Major ions concentration distribution along with sampling station.
